# Interplay between the Hsp90 Chaperone and the HslVU Protease To Regulate the Level of an Essential Protein in Shewanella oneidensis

**DOI:** 10.1128/mBio.00269-19

**Published:** 2019-05-14

**Authors:** Flora Ambre Honoré, Nathanael Jean Maillot, Vincent Méjean, Olivier Genest

**Affiliations:** a Aix Marseille Université, CNRS, BIP UMR 7281, IMM, Marseille, France; National Cancer Institute

**Keywords:** heat shock, proteases, protein chaperone, protein folding, proteostasis, stress adaptation

## Abstract

Protein synthesis, folding, and degradation are an accurately regulated process occurring in every organism and called proteostasis. This process is essential to maintain a healthy proteome since proteostasis dysregulation is responsible for devastating cellular issues. Proteostasis is controlled by a complex network of molecular chaperones and proteases. Among them, eukaryotic Hsp90, assisted by many cochaperones and the Hsp70 chaperone system, plays a major role in activating hundreds of client proteins, and Hsp90 inhibition usually leads to proteasomal degradation of these clients. In bacteria, however, the precise function of Hsp90 remains quite unclear, and only a few clients are known. Recently, we have shown that Hsp90 is essential at elevated temperature in the aquatic model bacterium Shewanella oneidensis, and we have identified a client of Hsp90, TilS, involved in tRNA modification. Here we found that two members of the proteostasis network with antagonist activities, the Hsp90 chaperone and the HslVU protease, which is considered the proteasome ancestor, together regulate the level of TilS. In particular, we show that deletion of the genes coding for the HslVU protease suppresses the growth defect of an S. oneidensis strain with *hsp90* deleted, by increasing the cellular level of the essential TilS protein. These results open up new avenues for understanding how proteostasis is controlled in bacteria, and new Hsp90 clients are much needed now to confirm the interplay between Hsp90 and proteases.

## Abstract (precis)

Maintaining a healthy proteome is essential in every living cell from bacteria to humans. For example, proteostasis (protein homeostasis) imbalance in humans leads to devastating diseases, including neurodegenerative diseases and cancers. Therefore, proteins need to be assisted from their synthesis to their native folding and ultimately to their degradation. To ensure efficient protein turnover, cells possess an intricate network of molecular chaperones and proteases for protein folding and degradation. However, these networks need to be better defined and understood. Here, using the aquatic bacterium Shewanella oneidensis as a model organism, we demonstrate interplay between two proteins with antagonist activities, the Hsp90 chaperone and the HslVU protease, to finely regulate the level of an essential client of Hsp90. Therefore, this work provides a new bacterial model to better study protein regulation and turnover, and it sheds light on how proteostasis by Hsp90 and proteases could be controlled in bacteria.

## OBSERVATION

Proteostasis is controlled in every organism by a complex network of chaperones and proteases (1, 2). Among them, the eukaryotic 90-kDa heat shock protein (Hsp90) chaperone, assisted by many cochaperones and the Hsp70 chaperone system, remodels and activates hundreds of client proteins, including kinases and receptors (3–7). In bacteria, Hsp90 also collaborates with the DnaK chaperone system, but cochaperones are absent, and its function needs to be clarified (8–13). In addition, only a few bacterial Hsp90 client proteins are known (8, 14–19). Using the aquatic proteobacteria Shewanella oneidensis, we have recently found that Hsp90 is necessary under heat stress conditions to protect and activate the TilS protein, leading to bacterial growth (17). TilS is an essential enzyme that modifies the specificity of the only tRNA that translates the AUG initiator codon in methionine into a tRNA that translates the AUA rare codon in isoleucine (20, 21). Given the major importance for whole proteome synthesis to correctly translate the AUG initiator codon, the level of TilS has to be finely regulated in the cell, since an excess of TilS could lead to depletion of the tRNA-AUG in favor of tRNA-AUA. On the other side, the absence of TilS prevents translation of proteins containing the AUA codon. Here we show that there is interplay between two components of the proteostasis network to regulate the level of the TilS protein: (i) the Hsp90 chaperone for protection and activation and (ii) the HslVU protease for degradation. Therefore, the level of TilS is precisely adjusted in the cell to allow correct protein translation and bacterial growth.

### The absence of HslVU suppresses the phenotype of the Δ*hsp90_So_* strain.

**FIG 1:**
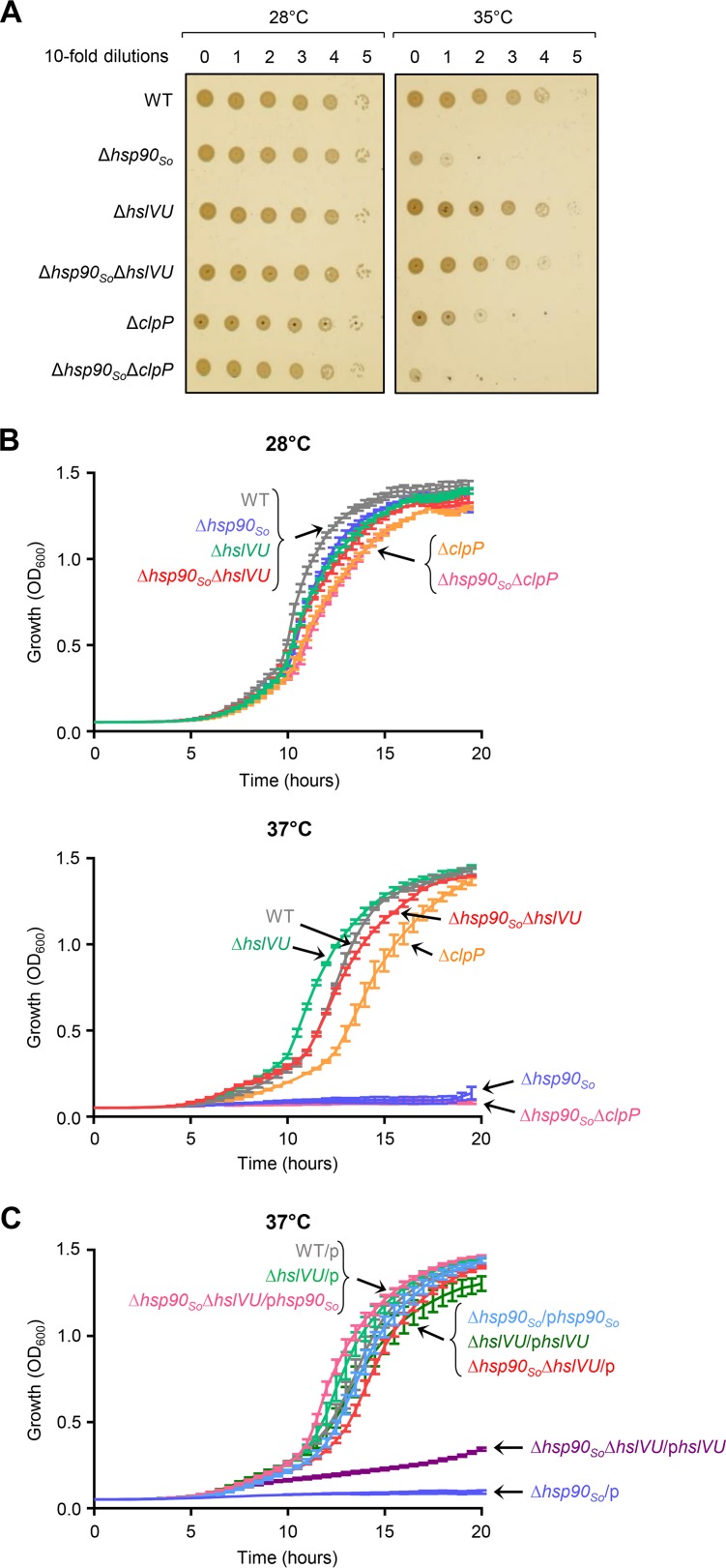
*hslVU* deletion suppresses the growth phenotype of the Δ*hsp90_So_* strain at high temperature. (A) Strains grown at 28°C to late exponential phase were diluted to an optical density at 600 nm (OD_600_) of 1, and 2 μl of 10-time serial dilutions was spotted onto LB agar plates. The plates were incubated at 28 or 35°C. (B) Strains grown at 28°C to late exponential phase were diluted to an OD_600_ of 0.0005 and incubated with shaking in a microplate reader at 28 or 37°C. (C) Strains were treated as in panel B, except that LB rich medium was supplemented with 0.015% arabinose to induce protein production from the plasmids. In panels B and C, data from at least three replicates are shown as mean ± standard error of the mean (SEM).

To get insight into how proteostasis is controlled in S. oneidensis during heat stress, we wanted to identify new components of the proteostasis network that could work with S. oneidensis Hsp90 (Hsp90_So_). As previously observed, we found that a strain with *hsp90_So_* deleted did not grow under heat stress conditions in liquid cultures and on solid media compare to the wild type (WT), whereas it grew well at the permissive temperature of 28°C (Fig. 1A and B) (17). We hypothesized that in the absence of Hsp90 at high temperature, one or several essential Hsp90_So_ clients are not correctly folded and are therefore degraded. Consequently, inactivation of the protease responsible for this degradation could restore a sufficient level of the client protein and could allow growth of the Δ*hsp90_So_* strain. We thus deleted the genes coding for the HslVU protease and for the ClpP subunit of the ClpAP and ClpXP proteases. These major proteolytic machines are composed of an ATP-dependent chaperone subunit belonging to the AAA+ family (i.e., HslU, ClpA and ClpX) that unfolds substrates and directs them into the catalytic chamber of the peptidase subunit (i.e., HslV and ClpP) (22). Strikingly, we observed that the absence of the HslVU protease did rescue the growth of the Δ*hsp90_So_* strain at high temperature in liquid and solid media (Fig. 1A and B, compare the Δ*hsp90_So_* Δ*hslVU* strain with the Δ*hsp90_So_* strain). In contrast, the ClpP protease did not seem to be involved in this pathway since no growth improvement was observed in the Δ*hsp90_So_* Δ*clpP* strain compared to the Δ*hsp90_So_* strain. At 28°C, all strains grew as wild type (Fig. 1A and B).

We then confirmed the growth phenotypes by producing HslVU or Hsp90_So_ from plasmids (Fig. 1C). We found that at high temperature, production of HslVU strongly reduced the growth of the Δ*hsp90_So_*Δ*hslVU* strain as expected, whereas production of Hsp90_So_ complemented the phenotype of the Δ*hsp90_So_* strain.

Altogether, these results support the idea that some essential Hsp90_So_ clients that are degraded in the Δ*hsp90_So_* strain are stabilized in the absence of HslVU. They therefore strongly suggest that these clients are degraded by the HslVU protease.

### The essential Hsp90_So_ client TilS is degraded by HslVU.

Since we have previously shown that the Hsp90_So_ client TilS is responsible for the growth defect of the Δ*hsp90_So_* strain at high temperature (17), we looked at its level under stress conditions with or without Hsp90_So_ and/or HslVU. To do that, a plasmid coding for TilS with a 6× His tag was introduced in the different genetic backgrounds of S. oneidensis. The strains were grown at sublethal high temperature, TilS expression was induced, and its amount was determined by Western blot analysis.

**FIG 2:**
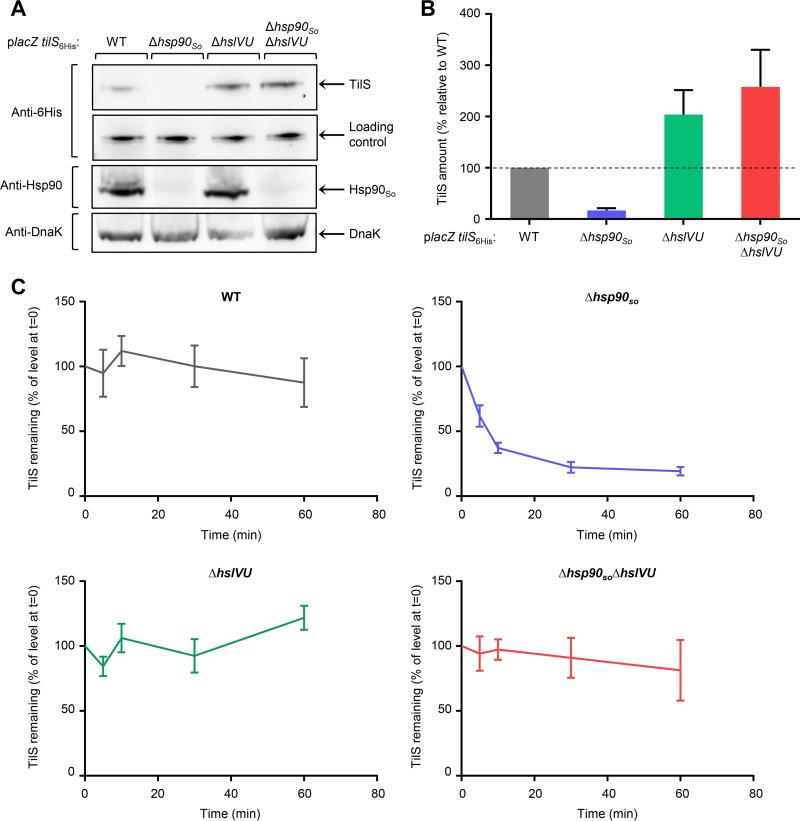
The HslVU protease degrades the Hsp90_So_ client TilS. (A) Strains containing the p*lacZ tilS_6His_* plasmid, in which *lacZ* and *tilS_6His_* are two independent genes under the control of the P_BAD_ promoter, were grown at 35°C, a sublethal temperature. At an OD_600_ of 0.6, 0.02% arabinose was added. After 2 h at 35°C, the same amounts of total protein extract from each strain were loaded for SDS-PAGE, transferred by Western blotting, and revealed with anti-6× His antibody to detect the TilS protein, anti-Hsp90 antibody, or anti-DnaK antibody. The loading control corresponds to a contaminating band revealed with the anti-6× His antibody, indicating that the same amount of cellular extracts was loaded. (B) Quantification of the amount of TilS was performed from 3 independent Western blots described in panel A, revealed with the anti-6× His antibody using ImageJ software. The amount of TilS measured in the wild-type strain was set to 100%. Data are shown as mean ± SEM. (C) Chase experiments. Strains containing the p*lacZ tilS_6His_* plasmid were grown as in panel A, except that 0.2% arabinose was added to increase the level of the TilS protein, in particular in the Δ*hsp90_So_* strain. After 2 h of induction, 200 μg/ml chloramphenicol was added to block protein translation (*t* = 0). Samples were taken at several times after chloramphenicol addition, and proteins were precipitated with trichloroacetic acid (TCA), loaded for SDS-PAGE, and quantified on a Western blot, revealed with anti-6× His antibody using the ImageJ software. The amount of TilS measured in each strain at *t* = 0 (chloramphenicol addition) was set to 100%. Data are shown as mean ± SEM.

As already seen, we found that about 85% of TilS was degraded in the Δ*hsp90_So_* strain compared to the wild type (Fig. 2A and B) (17). Interestingly, the TilS level was dramatically increased in the Δ*hsp90_So_* Δ*hslVU* strain, reaching more than 15 times the level observed in the absence of Hsp90_So_. These results strongly support the idea that TilS is degraded by the HslVU protease. In the Δ*hslVU* strain, we observed that the TilS level is higher than in the wild-type strain, suggesting that in the presence of Hsp90_So_, part of the pool of TilS is degraded by the HslVU protease. In addition, we found that most of TilS protein was present in the soluble fraction of the different strains (see Fig. S1A and B in the supplemental material).

10.1128/mBio.00269-19.2 FIG S1 TilS protein is mainly found in the soluble fraction. (A) Strains containing the p*lacZ tilS_6His_* plasmid were grown at 35°C, and 0.2% arabinose was added at an OD_600_ of 0.6 to induce TilS production. After 3 h, cells were lysed, and extracts were centrifuged to separate the soluble fraction (supernatant [S]) and the insoluble fraction (pellet [P]). Proteins from both fractions were loaded for SDS-PAGE, transferred by Western blotting, and revealed with an anti-6× His antibody to detect TilS. (B) Quantification of the TilS protein from the soluble (S) or insoluble (P) fraction. Amounts of TilS were determined with ImageJ software from three independent Western blots obtained as in panel A, except that the pellet fractions have been concentrated 10 times to observe a significant band, as shown in the inset. The total amount of TilS (soluble plus insoluble) from each strain was set to 100%. In the Δ*hsp90_So_* strain, the amount of TilS was too low to be accurately quantified and was therefore not determined (ND). Data from three replicates are shown as mean ± SEM. Download FIG S1, PDF file, 0.08 MB. Copyright © 2019 Honoré et al. 2019 Honoré et al. This content is distributed under the terms of the Creative Commons Attribution 4.0 International license.

As a control, we showed that transcription from this plasmid did not vary in the different strains grown at sublethal temperature (see Fig. S2 in the supplemental material). We also checked that deletion of *hsp90_So_* and/or *hslVU* did not modify the heat shock response by quantifying the level of DnaK, whose gene is under the control of the RpoH sigma factor. Indeed, no significant variation was observed in the four strains (Fig. 2A).

10.1128/mBio.00269-19.3 FIG S2 Deletion of *hsp90_So_* and/or *hslVU* does not significantly affect protein production from the p*lacZ tilS_6His_* plasmid. Strains containing the p*lacZ tilS_6His_* plasmid were grown at 35°C, a sublethal temperature. At an OD_600_ of 0.6, 0.02% arabinose was added. After 2 h at 35°C, β-galactosidase activity was measured and expressed as Miller units. Data from three replicates are shown as mean ± SEM. Download FIG S2, PDF file, 0.05 MB. Copyright © 2019 Honoré et al. 2019 Honoré et al. This content is distributed under the terms of the Creative Commons Attribution 4.0 International license.

Finally, chase experiments were performed to measure kinetic of TilS degradation in the different genetic backgrounds. Strains containing the plasmid coding for TilS with a 6× His tag were grown as described previously, and after induction, a high concentration of chloramphenicol was added to block translation. The amount of TilS was quantified at several time points after chloramphenicol addition and was expressed relative to the level observed at time zero (Fig. 2C; see Fig. S3 in the supplemental material). In the absence of Hsp90_So_, TilS was degraded with time, and its level reached a plateau after about 30 min, whereas low or no degradation was found in the wild-type, Δ*hslVU*, and Δ*hsp90_So_* Δ*hslVU* strains (Fig. 2C; Fig. S3). These experiments demonstrate that TilS is degraded by the HslVU protease in the absence of Hsp90.

10.1128/mBio.00269-19.4 FIG S3 Western blots used to quantify the chase experiments shown in Fig. 2C. Strains containing the p*lacZ tilS_6His_* plasmid were grown at 35°C, a sublethal temperature. At an OD_600_ of 0.6, 0.2% arabinose was added. After 2 h of induction, 200 μg/ml chloramphenicol was added to block protein translation (*t* = 0). Samples were taken at several times after chloramphenicol addition, and proteins were precipitated with TCA, loaded on SDS-PAGE, and quantified on a Western blot, revealed with anti-6× His antibody. The Western blots shown are representative of 3 independent experiments. Download FIG S3, PDF file, 0.1 MB. Copyright © 2019 Honoré et al. 2019 Honoré et al. This content is distributed under the terms of the Creative Commons Attribution 4.0 International license.

In this article, we found that the level of TilS is highly regulated at a posttranslational level by the Hsp90_So_ chaperone and the HslVU protease, and we show that growth of the Δ*hsp90_So_* strain at elevated temperature strongly depends on the amount of the TilS protein. Interestingly, this result is reminiscent of our previous work, in which overproduction of TilS led to growth of the Δ*hsp90_So_* strain at high temperature (17). Therefore, increasing the level of TilS by two opposite mechanisms, overproduction or inactivation of the degradation, did result in the rescue of the phenotype of the Δ*hsp90_So_* strain. This finding reinforces the notion that the level of TilS needs to be tightly controlled.

Interestingly, interplay between Hsp90 and HslVU has already been proposed for the posttranslational regulation of an unknown protein involved in the synthesis of toxins in extraintestinal pathogenic Escherichia coli (23). In addition, the level of the Cas3 protein, a client of E. coli Hsp90, is reduced in the absence of Hsp90 in E. coli; however, the protease involved in the degradation has not yet been identified (15). This thus suggests that the antagonist activities of Hsp90 and HslVU could serve as a general mechanism to control the level of some proteins. In eukaryotes, connections between folding by the Hsp90 chaperone and degradation by the proteasome have been well established (24, 25). It therefore becomes essential to identify new clients of bacterial Hsp90 to confirm this model.

10.1128/mBio.00269-19.1 TEXT S1 Supplemental methods (growth conditions, strains, plasmids, bacterial growth, visualization and quantification of proteins, β-galactosidase assays, and chase experiments) and supplemental references. Download Text S1, DOCX file, 0.03 MB. Copyright © 2019 Honoré et al. 2019 Honoré et al. This content is distributed under the terms of the Creative Commons Attribution 4.0 International license.
