# Supplementary figures and images for: Interplay between the Hsp90 Chaperone and the HslVU Protease To Regulate the Level of an Essential Protein in Shewanella oneidensis
Source: mBio. 2019 May 14;10(3):e00269-19. doi: 10.1128/mBio.00269-19 (PMC6520445; doi:10.1128/mBio.00269-19)

Figure S1

A

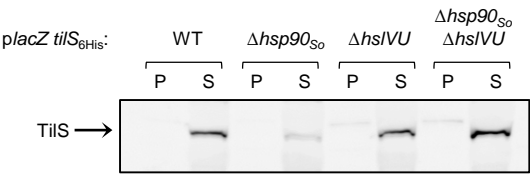

B

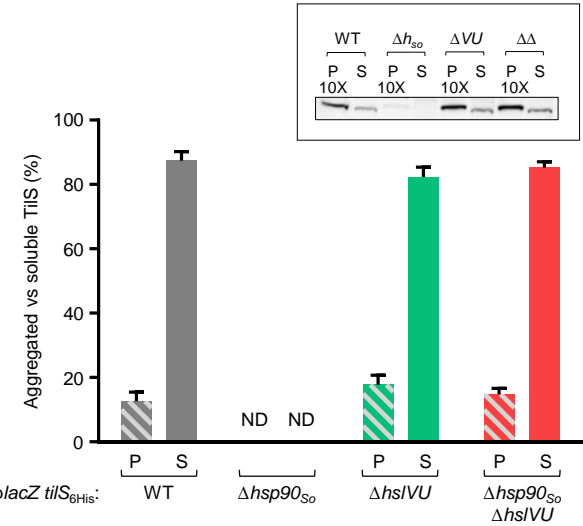

Supplement: FIG S1 [file mBio.00269-19-sf001.pdf]

Figure S2

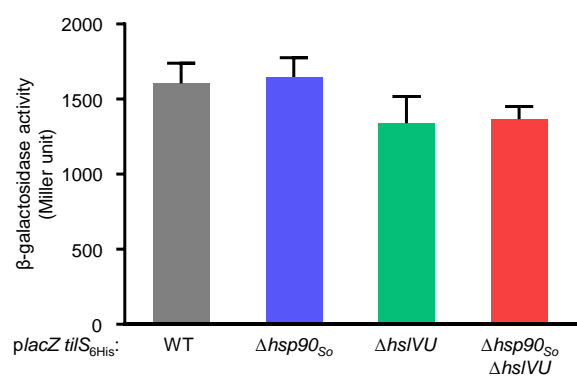

Supplement: FIG S2 [file mBio.00269-19-sf002.pdf]

Figure S3

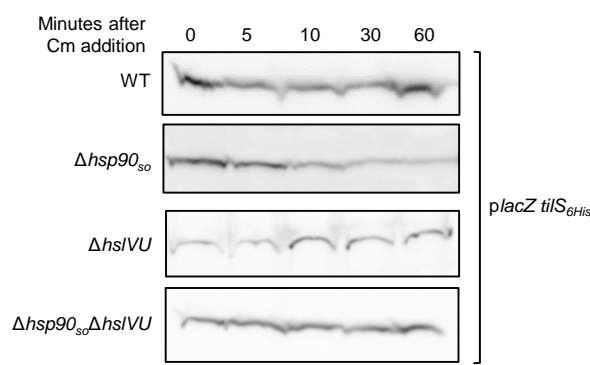

Supplement: FIG S3 [file mBio.00269-19-sf003.pdf]
