# Supplementary material for: Interplay between the Hsp90 Chaperone and the HslVU Protease To Regulate the Level of an Essential Protein in Shewanella oneidensis
Source: mBio. 2019 May 14;10(3):e00269-19. doi: 10.1128/mBio.00269-19 (PMC6520445; doi:10.1128/mBio.00269-19)
Supplement: TEXT S1 [file mBio.00269-19-s0001.docx]

**Supplemental methods**

**Growth conditions, strains and plasmids.** Strains were grown in rich LB medium with shaking at the temperatures indicated in the figures. When necessary, chloramphenicol (25 µg/mL) was added.

The wild-type *S. oneidensis* strain used in this study is MR1-R (1). Construction of the ∆*hsp90_So_* and ∆*clpP* strains have been described before (2, 3). The ∆*hslVU* strain was constructed by homologous recombination as described before (2, 4). Briefly, downstream and upstream 500 bp flanking regions of the *hslVU* genes (SO_4162 and SO_4163) were cloned at the restriction sites BamHI and SpeI into the suicide pKNG101 vector. The resulting plasmid was introduced into the *E. coli* strain CC118λpir (5), and transferred into MR1-R by conjugation. After integration, it was removed by adding 6% sucrose. Deletion of the *hslV and hslU* genes was confirmed by sequencing.

The ∆*hsp90_So_*∆*hslVU* and ∆*hsp90_So_*∆*clpP* strains were constructed by introducing by conjugation the pKNG101 plasmid containing downstream and upstream 500 bp flanking regions of the *hsp90_So_* gene (SO_2016) (2) into the ∆*hslVU* and ∆*clpP* strains, respectively. After integration, it was removed by adding 6% sucrose. Deletion of the *hsp90_So_* gene in the ∆*hslVU* and ∆*clpP* strains was confirmed by sequencing.

To construct the p*hslVU* plasmid, the coding sequence of *hslVU* with its physiological Shine-Dalgarno sequence was PCR-amplified from the chromosome of MR1-R with forward and reverse oligonucleotides containing the XmaI and XbaI restriction sites, respectively. After digestion with XmaI and XbaI, the DNA fragment was inserted into the pBad33 vector digested with the same enzymes. The p*hsp90_So_* (2) and p*lacZ* *tilS_6His_* plasmids (2) have already been constructed. Plasmids were introduced into *S. oneidensis* strains by conjugation.

**Bacterial growth.** After overnight growth at 28°C, cells were diluted to OD_600_=0.1 and incubated at 28°C. To follow bacterial growth on solid medium, cells in late exponential phase were diluted to OD_600_=1 and 2 µL of 10-fold serial dilutions were spotted on LB-agar plates that were subsequently incubated at 28°C or 35°C. To follow bacterial growth in liquid medium, cells in late exponential phase were diluted to OD_600_=0.0005 and incubated under shaking in a Tecan Spark microplate reader set at 28°C or 37°C. When necessary, arabinose was added as indicated in the figure legends.

**Visualization, quantification of proteins and β-galactosidase assays**. Strains containing the p*lacZ* *tilS_6His_* plasmid was grown overnight at 28°C. The cells were then diluted to OD_600_=0.1 and incubated at 35°C. At OD_600_=0.6, 0.02% arabinose was added, and cells were incubated for 2 more hours. Total protein extracts from the same amount of cells were heat-denatured, loaded on SDS-PAGE, transferred by Western blot and revealed with anti-6His (Invitrogen) antibody to detect the level of TilS, anti Hsp90_Ec_ (6), or anti-DnaK (produced by GenScript). The level of the TilS protein was quantified from three independent Western blots revealed with the anti-6His antibody using the ImageJ software.

To test if TilS is in the soluble or insoluble fraction, strains grown overnight at 28°C were diluted to OD_600_=0.1 and incubated at 35°C. At OD_600_=0.6, 0.2% arabinose was added and cells were collected 3 hours later. Cells were resuspended in 1x PBS, 1 mg/mL lysozyme and 20% sucrose, incubated at 4°C for 30’, then diluted 4 times in 1x PBS, lysed with French Press and centrifuged 15’ at 5,000 rpm. The supernatant (1 mL) was centrifuged 20’ at 13,000 rpm to separate the soluble (supernatant) and insoluble (pellet) fractions. Pellets were resuspended either in 1 mL of 1X PBS or in 100 µL (10x concentrated pellet fraction). Heat-denatured samples from the supernatant and pellet fractions were loaded on SDS-PAGE, transferred by Western blot and revealed with anti-6His (Invitrogen) antibody to detect TilS. The level of TilS was quantified from three independent Western blots using the ImageJ software.

To show that the same amount of protein was produced from the p*lacZ tilS_6His_* plasmid in the different genetic backgrounds, β-galactosidase activity was measured after 2 hours of induction with 0.02% arabinose.

**Chase experiments.** Strains containing the p*lacZ* *tilS_6His_* plasmid was grown overnight at 28°C. Cells were then diluted to OD_600_=0.1 and incubated at 35°C. At OD_600_=0.6, 0.2% arabinose was added, and cells were incubated for 2 more hours. At t=0, 200 µg/ml chloramphenicol was added to block protein synthesis. Samples were taken at t=0’, 5’, 10’, 30’ and 60’. Proteins were precipitated using TCA, heat-denatured, loaded on SDS-PAGE, transferred by Western blot and revealed with anti-6His (Invitrogen) antibody to detect the TilS protein. The level of TilS was quantified from three independent Western blots revealed with the anti-6His antibody using the ImageJ software.

**Supplemental references**

1. Bordi C, Iobbi-Nivol C, Méjean V, Patte J-C. 2003. Effects of ISSo2 insertions in structural and regulatory genes of the trimethylamine oxide reductase of *Shewanella oneidensis*. J Bacteriol 185:2042–2045.

2. Honoré FA, Méjean V, Genest O. 2017. Hsp90 Is Essential under Heat Stress in the Bacterium *Shewanella oneidensis*. Cell Rep 19:680–687.

3. Bouillet S, Genest O, Méjean V, Iobbi-Nivol C. 2017. Protection of the general stress response σS factor by the CrsR regulator allows a rapid and efficient adaptation of *Shewanella oneidensis*. J Biol Chem 292:14921–14928.

4. Baraquet C, Théraulaz L, Iobbi-Nivol C, Méjean V, Jourlin-Castelli C. 2009. Unexpected chemoreceptors mediate energy taxis towards electron acceptors in *Shewanella oneidensis*. Mol Microbiol 73:278–290.

5. Herrero M, de Lorenzo V, Timmis KN. 1990. Transposon vectors containing non-antibiotic resistance selection markers for cloning and stable chromosomal insertion of foreign genes in gram-negative bacteria. J Bacteriol 172:6557–6567.

6. Genest O, Reidy M, Street TO, Hoskins JR, Camberg JL, Agard DA, Masison DC, Wickner S. 2013. Uncovering a region of heat shock protein 90 important for client binding in *E. coli* and chaperone function in yeast. Mol Cell 49:464–473.
